# Frequency and genotypes of *Chlamydia trachomati*s in patients attending the obstetrics and gynecology clinics in Jalisco, Mexico and correlation with sociodemographic, behavioral, and biological factors

**DOI:** 10.1186/s12905-017-0428-5

**Published:** 2017-09-15

**Authors:** Néstor Casillas-Vega, Rayo Morfín-Otero, Santos García, Jorge Llaca-Díaz, Eduardo Rodríguez-Noriega, Adrián Camacho-Ortiz, Ma de la Merced Ayala-Castellanos, Héctor J. Maldonado-Garza, Jesús Ancer-Rodríguez, Guadalupe Gallegos-Ávila, Alberto Niderhauser-García, Elvira Garza-González

**Affiliations:** 1 Departamento de Patología Clínica, Hospital Universitario Dr. Jose Eleuterio Gonzalez, Universidad Autónoma de Nuevo León, Monterrey, Nuevo León Mexico; 2 Servicio de Gastroenterología, Hospital Universitario Dr. Jose Eleuterio Gonzalez, Universidad Autónoma de Nuevo León, Av. Gonzalitos and Madero, Mitras Centro PC, 64460 Monterrey, Nuevo León Mexico; 3 Hospital Civil de Guadalajara, Fray Antonio Alcalde, y el Instituto de Patología Infecciosa y Experimental, Centro Universitario de Ciencias de la Salud, Universidad de Guadalajara, Guadalajara, Jalisco Mexico; 4 Departamento de Microbiología e Inmunología, Facultad de Ciencias Biológicas Universidad Autónoma de Nuevo León, San Nicolás, Nuevo León Mexico; 5 Coordinación de Epidemiología Hospitalaria, Hospital Universitario Dr. Jose Eleuterio Gonzalez, Universidad Autónoma de Nuevo León, Monterrey, Mexico; 6 Departamento de Patología, Facultad de Medicina, Universidad Autónoma de Nuevo León, Monterrey, Nuevo León Mexico

**Keywords:** *Chlamydia trachomatis*, Genotype, Pregnancy, Mexico, Lymphogranuloma venereum

## Abstract

**Background:**

*Chlamydia trachomatis* is the causative agent of the most common bacterial sexually transmitted infection worldwide. The aim of this study was to investigate the frequency and genotypes of *C. trachomati*s in patients attending an obstetrics and gynecology clinic in Jalisco, Mexico and correlates them with sociodemographic, behavioral, and biological factors.

**Methods:**

*C. trachomatis* detection was performed in endocervical samples from 662 patients by direct fluorescence assay (DFA) and two PCR assays that amplified the phospholipase D endonuclease superfamily protein (PLDESP) and *OmpA* genes. Positive samples were genotyped using PCR–restriction fragment length polymorphism assays. Sociodemographic, behavioral, and biological data were collected.

**Results:**

The mean age of the study population was 31 (range, 14–78) years. *C. trachomatis* positivity was detected by DFA in 16.7% (*n* = 111), PLDESP gene amplification in 14.2% (*n* = 94), and *OmpA* gene amplification in 14.5% (*n* = 96) of the population. Eight *C. trachomatis* genotypes were detected: E (39.6%), F (29.2%), D (15.6%), K (6.3%), L2 (3.1%), G, J, and I (2.1% each). *C. trachomatis* infection was associated with age, marital status, pregnancy, and hormonal contraceptive use (all *p* = 0.01); intrauterine device use and previous premature birth (both *p* = 0.03); and infection during pregnancy, previous ectopic pregnancy, pelvic inflammatory disease **(**PID), and green vaginal discharge (all *p* = 0.04)*. C. trachomatis* genotype K was more likely to be detected in women histories of ≥2 sexual partners, genotype F was more likely in pregnant women, genotype L2 was more likely in women with PID, genotype D was more likely in women who had had infection during previous pregnancies, and genotype E was more likely in those with previous ectopic pregnancies and green vaginal discharge (all *p* = 0.01).

**Conclusions:**

The frequency of *C. trachomatis* in our population was higher than previously reported worldwide, but within the range reported for Mexico. Genotype E was detected most frequently in the study population. Infection by *C. trachomatis* and *C. trachomatis* genotypes K, F, D, and E was strongly associated with multiple sociodemographic, behavioral, and biological factors. *C. trachomatis* genotype L2 was detected in women with PID.

## Background

*Chlamydia trachomatis* is the causative agent of the most common bacterial sexually transmitted infection (STI) worldwide [1], and the most frequently reported of all notifiable infections in the United States and Europe. In the United States alone, an estimated 2 – 5 million new cases of *C. trachomatis* infection occur each year [2, 3]. The infection is asymptomatic in almost 80% of women, and untreated genital infections lead to serious complications, such as pelvic inflammatory disease (PID) and infertility [4]. Asymptomatic infected people not only are at risk of developing serious long-term sequelae, but also transmit the infection. In these individuals, diagnosis of *C. trachomatis* infection is difficult because the pathogen load is low [5].

Nucleic acid amplification tests are the preferred method for the diagnosis of *C. trachomatis* genital infection because of their high sensitivity and specificity [4]. *C. trachomatis* typing is based on assays of the major outer membrane protein (MOMP). This analysis differentiates 18 genotypes based on changes in the single copy of the *OmpA* gene, which encodes the MOMP [6]. Genotypes A to C are associated with trachoma, genotypes D to K are associated with common urogenital and ocular pathogens in adults, and genotypes L1 to L3 are associated with lymphogranuloma venereum (LGV). In developed countries, genotypes D, E, and F are most commonly associated with genital infections [7–9].

Previous studies have documented *C. trachomatis* frequencies ranging from 1.5 to 28% in female populations in Mexico [10, 11]. The only Mexican study that has included reporting on *C. trachomatis* genotypes showed that 24 of 152 (15.8%) infertile patients were positive for *C. trachomatis*, and that the most prevalent genotype was F [12]. Thus, additional studies describing the frequency and genetic distribution of *C. trachomatis* in Mexico are needed. The aim of this study was to investigate the frequency and genotypes of *C. trachomati*s in patients attending the obstetrics and gynecology clinic of the Fray Antonio Alcalde Civil Hospital in Jalisco, Mexico and correlates them with Sociodemographic, behavioral, and biological factors.

## Methods

### Ethics statement

This study was performed with the approval of the Local Ethics Committee of the Hospital Civil de Guadalajara “Fray Antonio Alcalde” in Jalisco, Mexico (approval no. 062/13). Written informed consent was obtained from all patients or, for minors, from caretakers or guardians.

### Study population and data collection

This was a cross-sectional study, conducted in the obstetrics and gynecology department of the Hospital Civil de Guadalajara “Fray Antonio Alcalde” from September 2013 to August 2014. Only patients attending the clinic for the first time were invited to participate. Sociodemographic and clinical data were collected for each patient.

### Specimen collection and DNA isolation

Endocervical samples were collected using two sterile Dacron-tipped swabs and placed in 2-sucrose phosphate medium. Tubes were stored at −20 °C until analyzed.

To obtain *C. trachomatis* DNA, one swab was subjected to enzymatic lysis with lysozyme and proteinase K. Genomic DNA was extracted using a standard phenol-chloroform-isoamyl alcohol protocol. The concentration and purity of the extracted DNA was evaluated through a spectrophotometer (GENESYS™ 20, Thermo Fisher Scientific, Germany).

### Direct fluorescence assay

*C. trachomatis* was detected by direct fluorescence assay (DFA) using a monoclonal anti-*Chlamydia* antibody (BioMérieux, France). The endocervical samples were transferred to clean slides, which were incubated with fluorescent monoclonal antibody for 30 min at a controlled temperature of 25 °C. The samples were observed under a fluorescence microscope (Axio Scope.A1; Zeiss, NY). Positive and negative controls were included in all assays.

### Phospholipase D Endonuclease Superfamily protein gene analysis

Polymerase chain reaction (PCR) assays were performed to amplify the phospholipase D endonuclease superfamily (PLDESP) gene using the method described by Gimenes et al. [13] and amplify a fragment of 361 bp for about 60 min. The total volume was 25 μL, consisting of 2.5 μL 10× PCR buffer, 100 ng DNA, 3 mM MgCl_2_, 200 μM of each deoxynucleotide triphosphate (dNTP), and 1 U Taq polymerase (Bioline, London, UK). The primers used were CT-F (5′-TCTTTTTAAACCTCCGGAACCCACTT-3′) and CT-R (5′-GGATGGCATCGCATAGCATTCTTTG-3′). Each run was accompanied by positive and negative control sample. The thermocycler conditions were: 3 min at 94 °C; then 35 cycles of 30 s at 94 °C, 30 s at 50 °C, and 30 s at 72 °C; and a final extension of 5 min at 72 °C. The amplification products were visualized using electrophoresis on a 2.5% agarose gel stained with 1 μg/mL ethidium bromide, and compared with a standard (DNA Molecular Weight, Marker 100, Sigma Aldrich, St. Louis, MO, USA), and developed with a UV transilluminator (UVP BioImaging Systems Photodocumentator, Upland, CA).

### *OmpA* gene analysis and restriction fragment length polymorphism analysis

The *OmpA* gene of *C. trachomatis* was amplified using the method described by Lan et al. [14] with small modifications, to amplify a fragment of 1100 bp The total volume was 25 μL, consisting of 2.5 μL 10× PCR buffer, 100 ng DNA, 3 mM MgCl_2_, 200 μM of each dNTP, and 1 U Taq polymerase (Bioline). The primers used were SERO1A (5′-ATGAAAAAACTCTTGAAATCGG-3′) and SERO2A (5′-TTT CTA GAT CTT CAT TCT TGT T-3′). Each run was accompanied by positive and negative control sample. The thermocycler conditions were: 7 min at 94 °C; then 40 cycles of 3 min at 95 °C, 3 min at 45 °C, and 3 min at 72 °C; and a final extension of 7 min at 72 °C. The amplification products were visualized using electrophoresis on a 1.5% agarose gel stained with 1 μg/mL ethidium bromide, and compared with a standard (DNA Molecular Weight, Marker 1000, Sigma Aldrich, St. Louis, MO, USA), and developed with a UV transilluminator (UVP BioImaging Systems Photodocumentator, Upland, CA). Then, 0.5 μl of the first-round PCR product was used for semi-nested PCR, which was performed with the same reagents and conditions except for the primers. The primers used for this assay were SERO2A and the nested primer PCTM3 (5′-TCCTTGCAAGCTCTGCCTGTGGGGAATCCT-3′). The PCR products of the second round were analyzed using electrophoresis on a 1.5% agarose gel stained with 1 μg/mL ethidium bromide, and compared with a standard (DNA Molecular Weight, Marker 1000, Sigma Aldrich, St. Louis, MO, USA), and developed with a UV transilluminator (UVP BioImaging Systems Photodocumentator, Upland, CA). Positive samples were genotyped by PCR–restriction fragment length polymorphism (RFLP) assays, as described by Sayada et al. [15], using 1000-bp semi-nested PCR products. The nested PCR product (10 μL) was digested with 2.5 U *Alu* I (Promega, Madison, WI, USA) overnight at 37 °C. Depending on the patterns obtained, the products were digested with the enzyme *Hinf* I (Promega). Genotypes were identified by their restriction patterns on ethidium bromide–stained 3.5% agarose gels. We used control DNA from the *C. trachomatis* strain ATCC VR-902B MOMP gene, partial cds (AmpliRun, ref. MBC012).

### Statistical analysis

Descriptive analysis was performed. We used the Wilcoxon rank-sum test to compare mean values and Fisher’s exact test or the chi-squared test to compare dichotomous variables. Analyses were performed using SPSS (version 18).

## Results

### Study population and patient characteristics

Of 30,000 female patients estimated who visited the obstetrics and gynecology department, 662 patients were enrolled in this study. Only patients who agreed to participate and were attending the clinic for the first time were included. The mean age of the study population was 31 (range, 14–78) years. The largest proportion (38.5%, *n* = 255) of patients was aged 20–29 years. Most (75.5%, *n* = 500) patients were married or living with a partner, and 37.6% (*n* = 249) reported using condoms as a contraceptive method. The majority (69.5%, *n* = 460) of patients were housewives, and 43.3% (*n* = 287) were pregnant at the time of sample collection (Table 1).

**Table 1:** Associations of *C. trachomatis* with sociodemographic, behavioral, biological factors and specific symptoms of the study population

Characteristic		Total positive population [*n* (%), *n* = 662]	*C. trachomatis* positive by *OmpA* [*n* (%), *n* = 96]	*P*	Adjusted OR (95% CI)
SOCIODEMOGRAPHIC FACTORS					
Age group (years)					
	14–19	93 (14)	10 (10.4)	0.14	0.68 (0.32, 1.33)
	20–29	255 (38.5)	35 (36.5)	0.32	0.90 (0.57, 1.41)
	30–39	186 (28.1)	23 (24)	0.16	0.77 (0.46, 1.27)
	≥ 40	129 (19.5)	28 (29.2)	0.01	2.69 (1.61, 4.44)
Occupation					
	Housewife	460 (69.5)	64 (66.7)	0.26	0.85 (0.53, 1.32)
	Employee/merchant	106 (16)	19 (19.8)	0.13	1.37 (0.77, 2.36)
	Professional	63 (9.5)	8 (8.3)	0.34	0.84 (0.36, 1.77)
	Student	33 (5)	5 (5.2)	0.43	1.05 (0.35, 2.66)
Marital status					
	Single	140 (21.2)	4 (4.2)	0.43	1.05 (0.32, 2.61)
	Divorced/widowed/separated	22 (3.3)	8 (8.3)	0.34	0.84 (0.36, 1.77)
	Married/living with partner	500 (75.5)	83 (86.5)	0.01	1.99 (1.10, 3.83)
BEHAVIORAL FACTORS					
Alcohol use		145 (21.9)	23 (24)	0.31	1.12 (0.69, 1.77)
Current smoker		200 (30.21)	31 (32.3)	0.29	1.14 (0.67, 1.89)
Age at first intercourse (years)					
	< 18	330 (49.9)	47 (49)	0.43	0.96 (0.62, 1.49)
	≥ 18	332 (50.2)	49 (51)	0.17	1.23 (0.79, 1.91)
No. of sex partners					
	0	7 (1)	2 (2.1)	0.16	2.38 (0.31,12.27)
	1	311 (47)	40 (41.7)	0.13	0.77 (0.49, 1.20)
	2–4	279 (42.2)	43 (44.8)	0.28	1.13 (0.73, 1.75)
	≥ 5	65 (9.8)	11 (11.5)	0.27	1.22 (0.59, 2.38)
Contraceptive use					
	Hormonal	102 (15.4)	7 (7.3)	0.01	0.39 (0.16,0.38)
	Condom	249 (37.6)	39 (40.6)	0.25	1.16 (0.74, 1.82)
	IUD	67 (10.1)	15 (15.6)	0.03	1.79 (0.93, 3.28)
	Bilateral tubal occlusion	40 (6)	5 (5.2)	0.37	0.83 (0.28, 2.06)
	None	204 (30.8)	39 (40.6)	0.02	1.55 (0.99, 2.42)
BIOLOGICAL FACTORS					
Pregnant at sampling		287 (43.3)	31 (32.3)	0.01	0.57 (0.36, 0.91)
Previous pregnancy		621 (93.8)	88 (91.7)	0.19	0.70 (0.32, 1.67)
Complications of previous pregnancy					
	Infection	386 (58.3)	49 (51)	0.04	0.69 (0.44, 1.07)
	Bleeding	69 (10.4)	10 (10.4)	0.48	0.99 (0.46, 1.97)
	Risk of spontaneous abortion	116 (17.5)	15 (15.6)	0.31	0.85 (0.45, 1.51)
	Premature birth	50 (7.5)	12 (12.5)	0.03	1.98 (0.96, 3.88)
	Ectopic pregnancy	9 (1.35)	4 (4.1)	0.03	3.46 (0.87,12.19)
Previous abortion		163 (24.6)	26 (27.1)	0.25	1.17 (0.71, 1.90)
SPECIFIC SYMPTOMS					
PID		295 (44.5)	36 (37.5)	0.04	0.68 (0.32, 1.33)
Dyspareunia					
	Pain /burning	222 (33.5)	27 (28.1)	0.11	0.74 (0.45, 1.19)
	Bleeding	28 (4.2)	3 (3.1%)	0.3	0.69 (0.16, 2.14)
Vaginal discharge					
Amount					
	Scarce	101 (15.2)	16 (16.7)	0.33	1.13 (0.61, 2.00)
	Moderate	322 (48.6)	44 (45.8)	0.27	0.87 (0.56, 1.35)
	Copious	185 (27.9)	26 (27.1)	0.42	0.95 (0.57, 1.53)
Color					
	White	450 (68)	65 (67.7)	0.47	0.98 (0.62, 1.58)
	Yellow	194 (29.3)	24 (25)	0.15	0.77 (0.46, 1.27)
	Green	45 (6.8)	11 (11.5)	0.04	1.90 (0.89, 3.81)
	Brown	19 (2.8)	5 (5.2)	0.47	0.98 (0.62, 1.58)
Previous infection					
	Urinary	188 (28.4)	23 (24)	0.14	0.76 (0.45, 1.25)
	Vaginal	420 (63.4)	58 (60.4)	0.25	0.86 (0.55, 1.34)

*OR* odds ratio, *CI* confidence interval, *IUD* Intrauterine Device, *PID* pelvic inflammatory disease

### Detection of infection

*C. trachomatis* positivity was detected by DFA in 16.7% (*n* = 111) of the study population, by PLDESP gene amplification in 14.2% (*n* = 94), and by *OmpA* gene amplification in 14.5% (*n* = 96) of the population. According to PCR-RFLP results, the most frequent *OmpA* genotype corresponded to E (39.6%, *n* = 38), followed by F (29.2%, *n* = 28), D (15.6%, *n* = 15), K (6.3%, *n* = 6), and L2 (3.1%, *n* = 3). Genotypes G, I, and J were detected less frequently (2.1%, *n* = 2 each; Table 2). Final results were regarded as true positives if the semi-nested PCR was positive (gold standard). The overall agreement of DFA results with semi-nested PCR the validation parameters were as follows: sensitivity 96%, specificity 97.3%, negative predictive value 99.3%, positive predictive value 86.5%, and accuracy 97.3%. Comparing the PCR results of the PLDESP gene with semi-nested PCR the validation parameters were as follows: sensitivity 98.6%, specificity 100%, negative predictive value 99.6%, positive predictive value 100%, and accuracy 99.7%.

**Table 2:** Associations of *C. trachomatis* genotypes with risk factors in the study population

Genotype	Total [*n* (%)]	≥2 sex partners			Pregnant at sampling			Pelvic inflammatory disease			Infection in previous pregnancy			Previous ectopic pregnancy			Green vaginal discharge		
		*n* (%)	*p*	Adjusted OR (95% CI)	*n* (%)	*p*	Adjusted OR (95% CI)	*n* (%)	*p*	Adjusted OR (95% CI)	*n* (%)	*p*	Adjusted OR (95% CI)	*n* (%)	*p*	Adjusted OR (95% CI)	*n* (%)	*p*	Adjusted OR (95% CI)
D	15 (15.6)	12 (80)	0	0.72 (0.18, 2.45)	4 (26.6)	0.3	0.72 (0.18, 2.45)	3 (20)	0.1	0.36 (0.07, 1.33)	4 (26.6)	0	0.29 (0.07, 0.9)	0 (0)	0.3	0 (0.0, 6.18)	0 (0)	0.1	0 (0.0, 1.63)
E	38 (39.6)	19 (50)	0	0.37 (0.01, 0.15)	13 (34.2)	0.4	1.154 (0.47, 2.79)	13 (34)	0.3	0.79 (0.33, 1.86)	20 (52.6)	0.4	1.11 (0.48, 2.54)	4 (10.5)	0	6.57 (0.79, 168.5)	9 (23.6)	0.01	7.67 (1.7, 55)
F	28 (29.2)	13 (46)	0	0.02 (0.01, 0.12)	13 (46.4)	0	2.30 (0.94, 6.07)	14 (50)	0.01	2.07 (0.83, 5.18)	16 (57.1)	0.2	1.4 (0.57, 3.49)	0 (0)	0.1	0 (0.0, 2.69)	1 (3.5)	0.1	0.21 (0.01, 1.39)
G	2 (2.1)	1 (50)	0.4	0.77 (0.01, 30.8)	1 (50)	0.3	2.11 (0.05, 84.4)	0 (0)	0.5	0.82 (0.02, 11.19)	2 (100)	0.1	–	0 (0)	0.5	0 (0.0, 88.48)	1 (50)	0.1	8.05 (0.19, 330.7)
I	2 (2.1)	1 (50)	0.4	0.77 (0.01, 30.87)	0 (0)	0.5	1.04 (0.03, 14.13)	0 (0)	0.2	0 (0.0, 5.39)	2 (100)	0.1	–	0 (0)	0.5	0 (0.0, 88.48)	0 (0)	0.4	0 (0.0, 27.63)
J	2 (2.1)	0 (0)	0.1	0 (0.0, 2.49)	0 (0)	0.2	0 (0.0, 7.3)	0 (0)	0.2	0 (0.0, 5.79)	2 (100)	0.1	–	0 (0)	0.5	0 (0.0, 88.48)	0 (0)	0.4	0 (0.0, 27.63)
K	6 (6.3)	6 (100)	0	7.92 (0.91, 245.2)	0 (0)	0.2	0.38 (0.01, 3.62)	3 (50)	0.3	1.71 (0.28, 10.5)	1 (50)	0.5	0.95 (0.15, 5.38)	0 (0)	0.4	0 (0.0, 18.84)	0 (0)	0.2	0 (0.0, 5.23)
L2	3 (3.1)	2 (66.6)	0.4	1.57 (0.11, 47.58)	0 (0)	0.2	0 (0.0, 3.59)	3 (100)	0.01	8.38 (0.70, 288.2)	0 (0)	0.1	0 (0.0, 1.61)	0 (0)	0.4	0 (0.0, 46.62)	0 (0)	0.3	0 (0.0, 13.84)

*OR* odds ratio, *CI* confidence interval

#### Association of *C. trachomatis* infection with sociodemographic, behavioral, biological factors, and specific symptoms

*C. trachomatis* infection was associated with age ≥ 40 years, being married or living with a partner, and the use of hormonal contraceptives (all *p* = 0.01), as well as intrauterine device (IUD) use (*p* = 0.03) and no history of birth control use (*p* = 0.02). In addition, pregnant women (*p* = 0.01) and those who had had infections during pregnancy (*p* = 0.04) were more likely to be infected. Infected women were more likely to have previous histories of giving birth prematurely (p = 0.03), ectopic pregnancy, PID, or green vaginal discharge (all *p* = 0.04; Table 1).

#### Association of *C. trachomatis* genotypes with risk factors, and specific symptoms

Women with histories of two or more sexual partners were more likely to be infected with *C. trachomatis* genotype K (*p* = 0.01), whereas pregnant women were more likely to be infected with *C. trachomatis* genotype F (*p* = 0.03). Associations were detected between genotype F, L2 is associated with PID (*p* = 0.02), genotype D and infection during previous pregnancy (*p* = 0.02), and genotype E was associated with previous ectopic pregnancy (*p* = 0.04) and green vaginal discharge (*p* = 001; Table 2). We found no association of any *C. trachomatis* genotype with previous pregnancy, abortion, premature birth and specific symptoms (data not shown).

## Discussion

*C. trachomatis* is a causal factor of STI, the course of which is frequently asymptomatic. In this study, the frequency and genotypes of *C. trachomati*s in patients attending a gynecology clinic in the largest public hospital in the state of Jalisco were determined. To our knowledge, this work is the largest molecular epidemiological study of *C. trachomatis* frequency and genotypes in Mexico, and their associations with sociodemographic, behavioral, and biological factors.

The frequency of *C. trachomatis* detected by PCR in this study was 14.5%. Previous studies in Mexican populations have detected *C. trachomatis* at frequencies ranging from 1.5% to 28% [10, 11]. Our results thus fall within the reported range for Mexico. The frequency detected in the current study was higher than reported for Australia (4.9%) [16, 17], Spain (4%) [17], France (3.6%) [18], and the United Kingdom (3%) [19].

Current guidelines from the Centers for Disease Control and Infection recommend annual screening for this bacteria in sexually active women aged ≤ 25 years and in those aged >25 years who are at risk of infection [20]. In Mexico, *C. trachomatis* infection is not a notifiable disease; thus, routine testing is not mandatory. This situation makes the collection of reliable epidemiological data from throughout the country difficult.

The prevalence of *C. trachomatis* genotypes has been determined for several countries, but little such data are available for Latin America. In our study, the most frequent genotype was E (39.6%), followed by F (29.2%) and D (15.6%). In a previous study of *C. trachomatis* in Mexico, genotype F was most frequent (54.2%), followed by genotypes E, G, K, and L2 (8.7% each); genotypes D, F, and I were detected at a frequency of 4.2% each [12]. That study, however, involved the examination of only 152 samples from infertile women (with only 24 specimens positive for *C trachomatis*), and limited clinical data were reported that contrasted with our results, this may be due to the small sample analyzed by the previous study [12]. Our results are similar to those reported from other Latin America and other parts of the world. In a Brazilian study that included 141 women, the most frequent genotype detected was E (39.7%), followed by F (17.7%) and D (17%) [21]. In a Costa Rican study including 806 *C. trachomatis*–positive samples, genotype E was also most frequent (31%), followed by F and D (21% each) [22]. Genotype E was most frequent in a study including 81 women conducted in Argentina [23]. Similar frequencies have been reported in other parts of the world, including the Netherlands (E, 41.5%; F, 21.8%; D, 11.9%) [24], China (E, 37.2%; F, 31.3%) [25], and Alabama, United States (E, 29%; F and D, 19% each) [26].

In our population, genotype L2 was identified in three patients with no clinical data of LGV. This situation has been described previously in Mexico [12]. LGV is a sexually transmitted disease caused by *C. trachomatis* serotypes L1, L2, and L3. It probably affects both sexes equally, although it has been reported more frequently in men, in whom the early manifestations of the disease are more evident. Men often display the acute form of LGV, whereas it is often detected in women when late-stage complications develop [27]. Most cases in Europe and North America have been identified in men who have sex with men [28]. A small number of genotype L cases has been reported in heterosexuals in the United Kingdom and Europe, including seven women in the UK since 2004, but these cases appear to be linked to male bisexual partners or sexual contact with those returning from endemic areas [29].

Interestingly, patients who were married or living with a partner were most at risk of infection with *C. trachomatis* in the present study. This finding may be explained by the lack of contraceptive use or infection prevention in this population.

An association of hormonal contraceptive use with the risk of infection was detected in the present study, confirming the results of previous studies [30]. Hormonal contraception is associated with an increased rate of cervical colonization because estrogen supports bacterial growth [31].

Recent studies have shown that IUD use is safe for all women, including those at high risk of STIs [32]. In this study, patients using IUDs were more likely to be infected with *C. trachomatis* than were those who did not report IUD use, possibly due to the trauma caused by IUDs.

Of special concern is the strong association between *C. trachomatis* infection and pregnancy at the time of sampling. Prompt diagnosis in this population is a cornerstone for the prevention of infection in newborns [33].

In the current study, *C. trachomatis* infection was associated with PID. Several studies have demonstrated that women who test positive for C*. trachomatis* have an increased risk of PID throughout their reproductive life compared with women who test negative [34, 35]. PID has also been suggested to be associated with an increased risk of repeated infection [36].

Possible associations of *C. trachomatis* genotypes with specific clinical symptoms or the pathogenicity of the disease have not been described completely. In a previous study, *C. trachomatis* genotype K was associated with vaginal discharge [37]; in our sample, genotype K was detected more frequently in patients with multiple partners. Furthermore, genotype F was detected more frequently in pregnant women. *C. trachomatis* genotype F has been associated with reduced mucopurulent endocervical discharge [38] and inflammation in men, but not in women [39]. Women who reported abdominal pain were more likely to be infected with genotype F [40]. In this study, genotype E was associated with previous ectopic pregnancy and green vaginal discharge. In a previous study, genotype E was associated with conjunctivitis in neonates [23].

## Conclusion

To our knowledge, this study is the only molecular epidemiological research to date on the frequency and genotypes of *C. trachomatis*, and their associations with sociodemographic, behavioral, and biological factors, in women attending a gynecology and obstetrics clinic in Mexico. This is a preliminary report and should be done in a much larger cohort, with a greater number of positive samples for Chlamydia. The frequency of *C. trachomatis* in our population was higher than previously reported worldwide, but within the range reported for Mexico. Genotype E was detected most frequent in the study population.

## Abbreviations

CI: Confidence interval
DFA: Direct fluorescence assay
dNTP: Doxynucleotide triphosphate
IUD: Intrauterine device
LGV: Lymphogranuloma venereum
MOMP: Major outer membrane protein
OR: Odds ratio
PCR: Polymerase chain reaction
PID: Pelvic inflammatory disease
PLDESP: Phospholipase D endonuclease superfamily protein
RFLP: Restriction Fragment Length Polymorphism Analysis
STI: Sexually transmitted infection
